# Patterning of Endothelial Cells and Mesenchymal Stem Cells by Laser-Assisted Bioprinting to Study Cell Migration

**DOI:** 10.1155/2016/3569843

**Published:** 2016-10-19

**Authors:** Jean-Michel Bourget, Olivia Kérourédan, Manuela Medina, Murielle Rémy, Noélie Brunehilde Thébaud, Reine Bareille, Olivier Chassande, Joëlle Amédée, Sylvain Catros, Raphaël Devillard

**Affiliations:** ^1^Bioingénierie Tissulaire, INSERM U1026, Bordeaux, France; ^2^Département d'Ophtalmologie, Université de Montréal, Montréal, QC, Canada; ^3^Bioingénierie Tissulaire, Université de Bordeaux, Bordeaux, France; ^4^Service d'Odontologie et de Santé Buccale, CHU de Bordeaux, Bordeaux, France

## Abstract

Tissue engineering of large organs is currently limited by the lack of potent vascularization* in vitro*. Tissue-engineered bone grafts can be prevascularized* in vitro* using endothelial cells (ECs). The microvascular network architecture could be controlled by printing ECs following a specific pattern. Using laser-assisted bioprinting, we investigated the effect of distance between printed cell islets and the influence of coprinted mesenchymal cells on migration. When printed alone, ECs spread out evenly on the collagen hydrogel, regardless of the distance between cell islets. However, when printed in coculture with mesenchymal cells by laser-assisted bioprinting, they remained in the printed area. Therefore, the presence of mesenchymal cell is mandatory in order to create a pattern that will be conserved over time. This work describes an interesting approach to study cell migration that could be reproduced to study the effect of trophic factors.

## 1. Introduction

The* in vitro* reconstruction of large tissues and organs by tissue engineering is currently limited by the lack of an appropriate vascularization of those constructs [1, 2]. The limited diffusion of nutrients and oxygen in connective tissues is influenced by the nature of the extracellular matrix as well as by the cell density and metabolic activity [3]. It has been shown that the lack of vascularization of tissue-engineered bones leads to hypoxia and cell death after implantation [3, 4] and that bone-regenerative capacity of bone marrow stromal cells is improved when those are transplanted into a bone defect model with endothelial cells [5]. Nowadays, several strategies for enhancing vascularization are under investigation [6]. They include scaffold designed to promote angiogenesis [7, 8],* in vitro* prevascularization [9–12], and inclusion of angiogenic factors [13].* In vitro *prevascularization has shown to be efficient for improving tissue inosculation after grafting [3, 14]. However, with traditional approaches to create an* in vitro* capillary-like network, there is no control over the architecture of the network. Seeding of endothelial cells (ECs) leads to a random network without possible organization into a complex structure. This drawback could be overcome by using bioprinting [15], allowing controlling the location of cells and built complex organs.

In the present study, human umbilical vein endothelial cells (HUVECs) and human bone marrow mesenchymal stem/stromal cells (HBMSCs), either alone or together, were patterned on a type I collagen biopaper using laser-assisted bioprinting (LAB). We evaluated the migration of endothelial cells depending on distance with neighbor cell islets and the presence of coprinted HBMSCs, early after bioprinting.

## 2. Material and Methods

### 2.1. Ethic Statement

This study was approved by the local institutional review board and follows the tenets of the Declaration of Helsinki. Written information was delivered to the patients (HBMSCs) or the mother (HUVECs) for use of their cells in research.

### 2.2. Cell Isolation and Culture

HBMSCs were obtained from human bone marrow according to methods described previously [16]. Briefly, bone marrow was aspirated from the femoral diaphysis or iliac bone after obtaining consent from patients undergoing hip prosthesis surgery after trauma. A single-cell suspension was obtained by sequential passages of the aspirate through 16-, 18-, and then 21-gauge needles. After centrifugation, the pellet was cultured in Minimum Essential Medium Alpha Modification (*α*-MEM; Gibco, Thermo Fisher Scientific, Paisley, UK), supplemented with 10% fetal bovine serum (FBS; Lonza, Verviers, Belgium). Cells were incubated in a humidified atmosphere with 5% CO_2_ at 37°C [16].

HUVECs were isolated as described previously [17]. Cells were expanded in Iscove's Modified Dulbecco's Medium (IMDM; Gibco, Thermo Fisher Scientific) containing 20% FBS, 12 *μ*g/mL endothelial cell growth supplement, and 90 *μ*g/mL heparin (ECGS/H 0.4% (v/v); PromoCell, Heidelberg, Germany) in gelatin-coated (0.2%; Sigma-Aldrich, Saint Quentin Fallavier, France) cell-culture flasks. Passage-1 cells were transduced with a lentiviral vector codding for the tdTomato fluorescent protein [18, 19]. Transduced and untransduced cells were used as stated for each experiment. After printing, cells were cultured in a medium containing equal volumes of IMDM 20% FBS with ECGS/H and of *α*-MEM 10% FBS [20].

### 2.3. Statistical Analyses

Statistical analyses were performed by unpaired *t*-test, using the GraphPad Prism 5 software (GraphPad Software Inc., La Jolla, CA, USA). *∗∗∗* = *p* < 0.001, ns = *p* > 0.05.

### 2.4. Bioprinting Procedure

**Figure 1:**
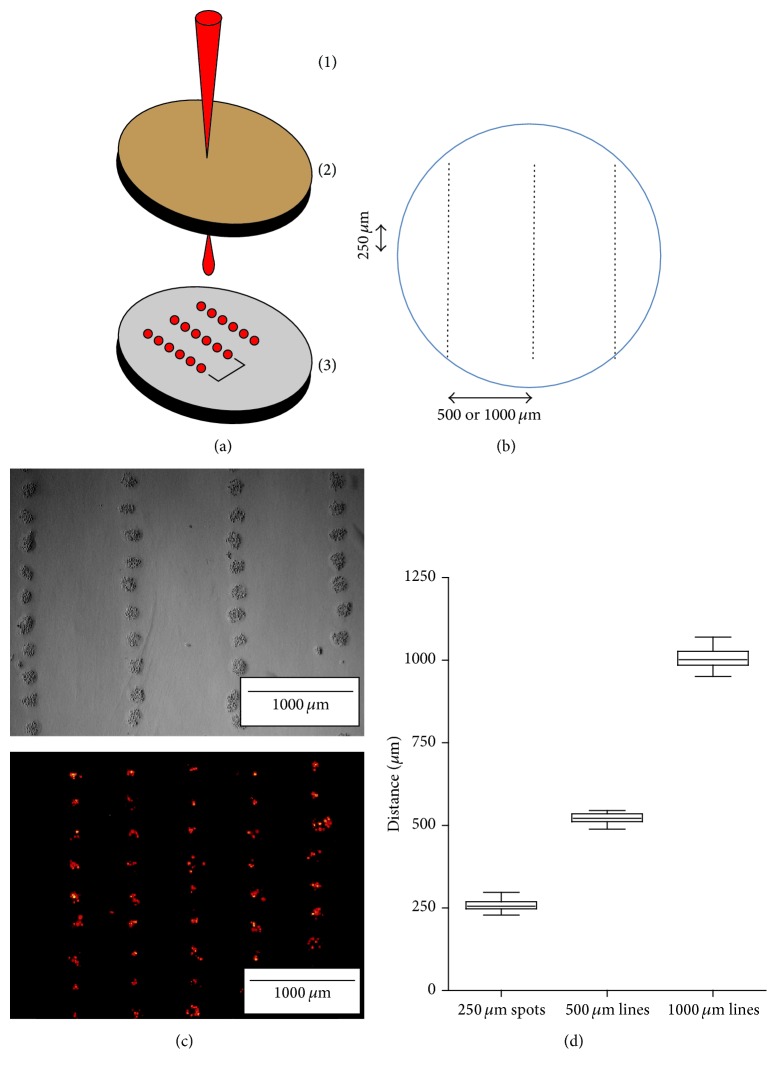
LAB setup and cell patterns. (a) The LAB setup: (1) laser beam, (2) donor gold-coated slide to generate the jet (the cell-containing solution is facing (3)), and (3) receptor collagen-coated slide. The red dots in (3) represent the cell islets on the collagen hydrogel postprinting. (b) Cell spots were printed at each 250 *μ*m in a given line segment and consecutive line segments were separated by either 500 *μ*m or 1000 *μ*m. (c) Examples of HBMSCs postprinting visualized by bright field view (laser energy 25 *μ*J) and tdTomato-expressing HUVECs in epifluorescence (laser energy 20 *μ*J). (d) Mean distances between spot centers (*n* = 60) and between segments (*n* = 30 for each distance), measured on the collector slide with ImageJ®.

The bioprinting procedure was performed as described previously in Guillotin et al. [21]. Briefly, two coplanar glass slides, one with the bioink and the other with the biopaper, were facing each other in the bioprinter. In order to transfer the laser energy to the bioink, the donor glass slide was coated with an energy-absorbing gold layer (60 nm) by plasma-enhanced sputter deposition (Emscope SC500; Elexience, Verrières-le-Buisson, France). Cultured HUVECs and HBMSCs were trypsinized and resuspended at 10^8^ cells/mL either alone (1) or together (1 : 1). 33 *μ*L of this cell suspension was spread on the glass slide (7 cm^2^), over the gold layer. The collector glass slide was covered with 141 *μ*L of a rat tail collagen type I solution (2 mg/mL; BD Biosciences, Bedford, MA), forming a 200 *μ*m thick layer. Laser pulses were focused on the gold layer and generated a jet that propels the cell suspension toward the collector slide (Figure 1(a)). Laser energy was adjusted regarding the cell type to obtain similar cell densities between mono- and coculture.

HUVECs and HBMSCs were printed, either alone (monoculture) or together (coculture), as lines (1000 *μ*m long) of cell-ink drops forming cell islets at each 250 *μ*m. Adjacent lines were either separated by 500 or 1000 *μ*m (Figures 1(b) and 1(c)). For each condition, distances between adjacent cell islets or lines were measured using ImageJ software (Figure 1(c)). HUVECs, expressing the tdTomato reporter gene, were printed alone at either 500 *μ*m or 1000 *μ*m of distance between adjacent lines. Cell tropism, either toward the formation of a continuous line or toward spreading, was monitored.

## 3. Results

The printing of HBMSCs and HUVECs allowed verifying the precision of the printing procedure. Printing the cells at a laser pulse repetition rate of 1 kHz allowed reaching an appropriate printing precision. The spot diameter was 170 ± 22 *μ*m, the interdot distance was 258 ± 16 *μ*m, and the interline distance was 520 ± 18 *μ*m or 1007 ± 28 *μ*m (mean ± SD) for 500 and 1000 *μ*m distances, respectively (Figure 1(d)). This data allows evaluating the precision of the laser bioprinter and determining that the standard error was ±20 *μ*m. This error was found to be sufficient for the present study. Therefore, a center-to-center distance of 250 *μ*m between spots was found to be appropriate to obtain a guided cell migration; a distance of 500 *μ*m between lines was selected to allow for migration between the lines in monoculture experiments.

**Figure 2:**
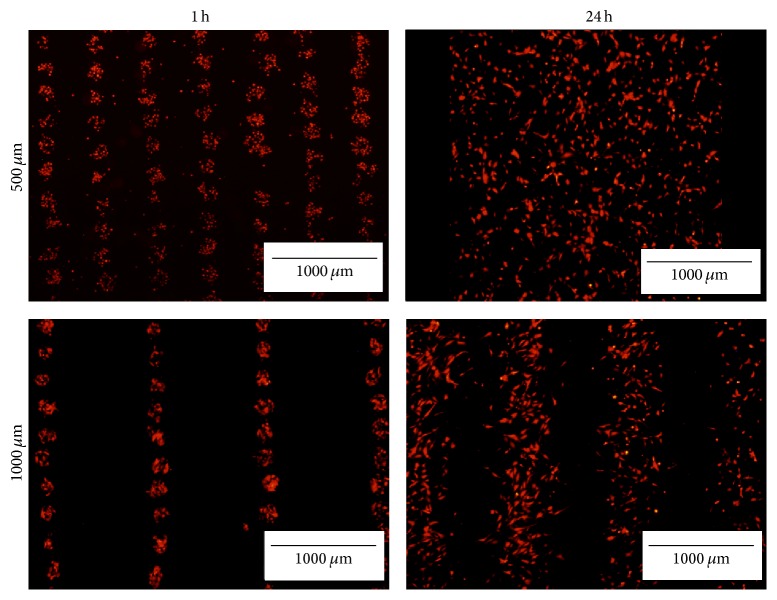
HUVECs spreading 24 hours after bioprinting. Comparison of the pattern of printed tdTomato HUVECs at 250 *μ*m between dots and either 500 or 1000 *μ*m between segments at 1 and 24 hours after printing. Images are representative of *n* = 3 experiments. Scale bar: 1000 *μ*m.

When imaged after 24 hours, tdTomato HUVECs printed with an interline distance of 500 *μ*m had spread homogeneously on the collagen hydrogel (Figure 2). When the distance was increased to 1000 *μ*m, the pattern was still discernable after 24 hours, despite cells' spreading (Figure 2). Therefore, when printed alone, HUVECs tend to migrate toward spreading on the collagen gel, regardless of the distance between the lines.

Bioprinting with the laser-assisted technology allowed us to easily form a pattern of ECs over a collagen matrix and evaluate if ECs display a preferential migration toward the closest cell islets or toward spreading uniformly. We observed that, in the absence of mesenchymal cells, endothelial cells migrated randomly on the matrix.

**Figure 3:**
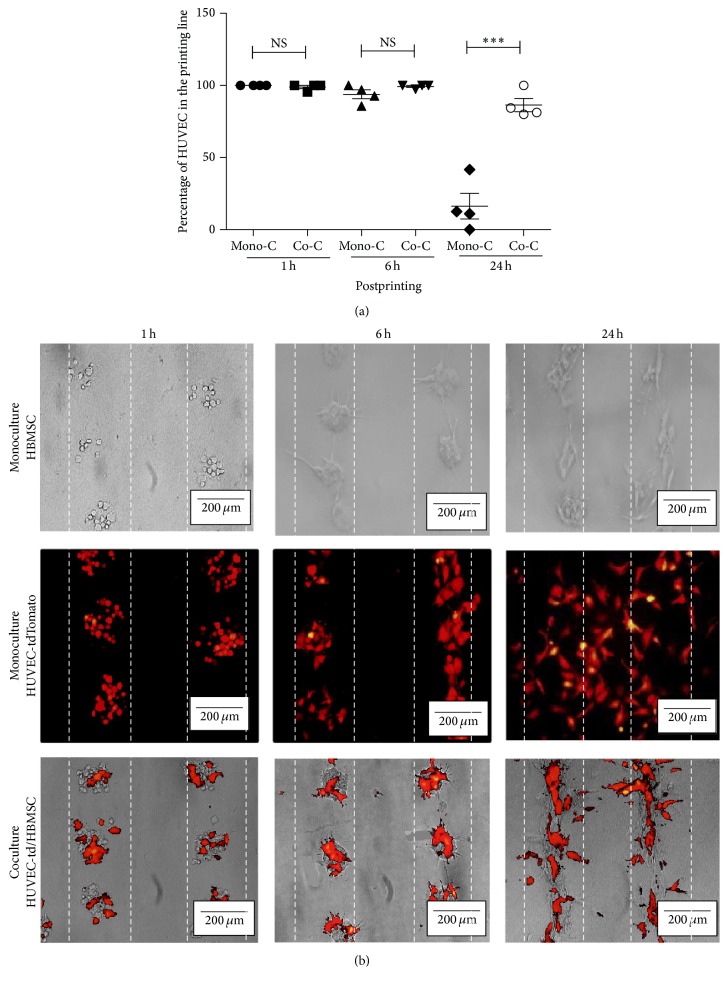
Distribution of HBMSCs and tdTomato HUVECs printed in monoculture versus coculture. (a) Percentage of HUVECs in the printing line at 1, 6, and 24 hours after bioprinting depending on the culture condition (mono/coculture) (^*∗∗∗*^ *p* < 0.001). (b) Representative images of follow-up over time of HBMSCs (upper line), tdTomato HUVECs (middle line), and tdTomato HUVECs-HBMSCs (lower line) at 1, 6, and 24 hours after printing, using an inverted microscope (Axiovert). In red: HUVECs, expressing tdTomato. Images were representative of *n* = 7 experiments. Distance between segments: 500 *μ*m.

When HBMSCs were printed alone with the same parameters (250 *μ*m × 500 *μ*m), they display a negligible migration on the collagen gel and tend to stay in lines for the first 24 hours (Figure 3). Interestingly, when cells were printed together to create a coculture (HUVECs tdTomato and HBMSCs, ratio 1 : 1), the HUVECs stay in the printing line instead of migrating in every direction (Figure 3).

## 4. Discussion

Our results indicate that coculture with mesenchymal cells is sufficient to allow for endothelial cells to stay in the printed area and to eventually form capillaries. This effect could be explained by the stabilization effect of MSC on capillaries [22, 23], which plays a role for the formation of a vascular network [24]. Indeed, the presence of mesenchymal cells printed together with endothelial cells allowed reducing endothelial cell migration. The long-term goal of the project presented here is to generate vascularized 3D bone tissue constructs with the ultimate goal of accelerating inosculation of the tissue-engineered graft. Control of the vascular network architecture is very important in the development of more complex bone tissues and being able to concentrate the ECs where they are needed is a precious advantage over random seeding of endothelial cells. Moreover, the precise positioning of other cell types is also possible with this bioprinting approach.

Canver et al. reported that the migration speed of a confluent monolayer of endothelial cells on collagen gel falls between 300 and 700 *μ*m for the first 24 hours, depending on the substrate stiffness [25]. In our study, cells migrated approximately 250 *μ*m in the same period. Differences may be attributable to the lower concentration of cells and to the bioprinting procedure.

The potential of endothelial cells to form tubular-like structures* in vitro* when cocultured with HBMSCs was demonstrated in multiple studies [22, 26–28]. However, the use of bioprinting to precisely deposit the cells into lines is scarce. Other groups have printed endothelial cells using laser-assisted bioprinting [29–31]. In Wu and Ringeisen's work, LAB was used to pattern HUVECs and human umbilical smooth muscle cells on Matrigel™ [32]. However, they did not study cell migration. Moreover, they used smooth muscle cells instead of BMSCs and Matrigel instead of collagen. In a recent publication by Takehara et al., ECs were patterned in a mesenchymal cell sheet using selective adhesion on the surface in order to generate a vascularized tissue with a precise architecture [33]. Even if their approach is similar to ours in terms of outcomes, bioprinting of the ECs is a more straightforward approach.

The use of LAB is particularly appropriate in order to create a 2D pattern of endothelial cell or coculture models on a collagenous extracellular matrix. Indeed, it allows precise printing of cells at high concentration without experiencing head-clogging problems. Printing at high cell concentration is important and allows a rapid formation of pseudocapillaries. Superposition of layers of collagen hydrogel with subsequent printing of endothelial cell patterns should allow the creation of a 3D construct.

This study shows that LAB is an appropriate tool for the deposition of ECs in tissue-engineered constructs and could be used to study cell migration in order to evaluate the influence of factors such as another cell type. It was demonstrated that mesenchymal stem cells have a great influence on ECs fate* in vitro*, by guiding their self-organization. This study sets the basis of ECs bioprinting on a collagenous hydrogel in coculture with mesenchymal cells. Future works will evaluate the behavior of the microvascular network on a longer time frame, including validation of the presence of a lumen in interconnecting capillaries. Furthermore, the capability to form 3D constructs will be evaluated by the superposition of multiple collagen layers with printed HUVECs and HBMSCs. Those studies will allow for the creation of tissue-engineered bone substitutes with precise microvascular-network architecture and therefore allow for a faster inosculation of the substitute following implantation and the reproduction of a physiological histoarchitecture.
